# Supplementary material for: Association between oral microbiome and seven types of cancers in East Asian population: a two-sample Mendelian randomization analysis
Source: Front Mol Biosci. 2023 Nov 21;10:1327893. doi: 10.3389/fmolb.2023.1327893 (PMC10702768; doi:10.3389/fmolb.2023.1327893)
Supplement: Supplementary file 1 [file DataSheet1.ZIP › Supplementary Materials/MR plots for tongue/tongue═╝/Colorectal cancer/pheno.267_to_colorectal cancer_forest.pdf]

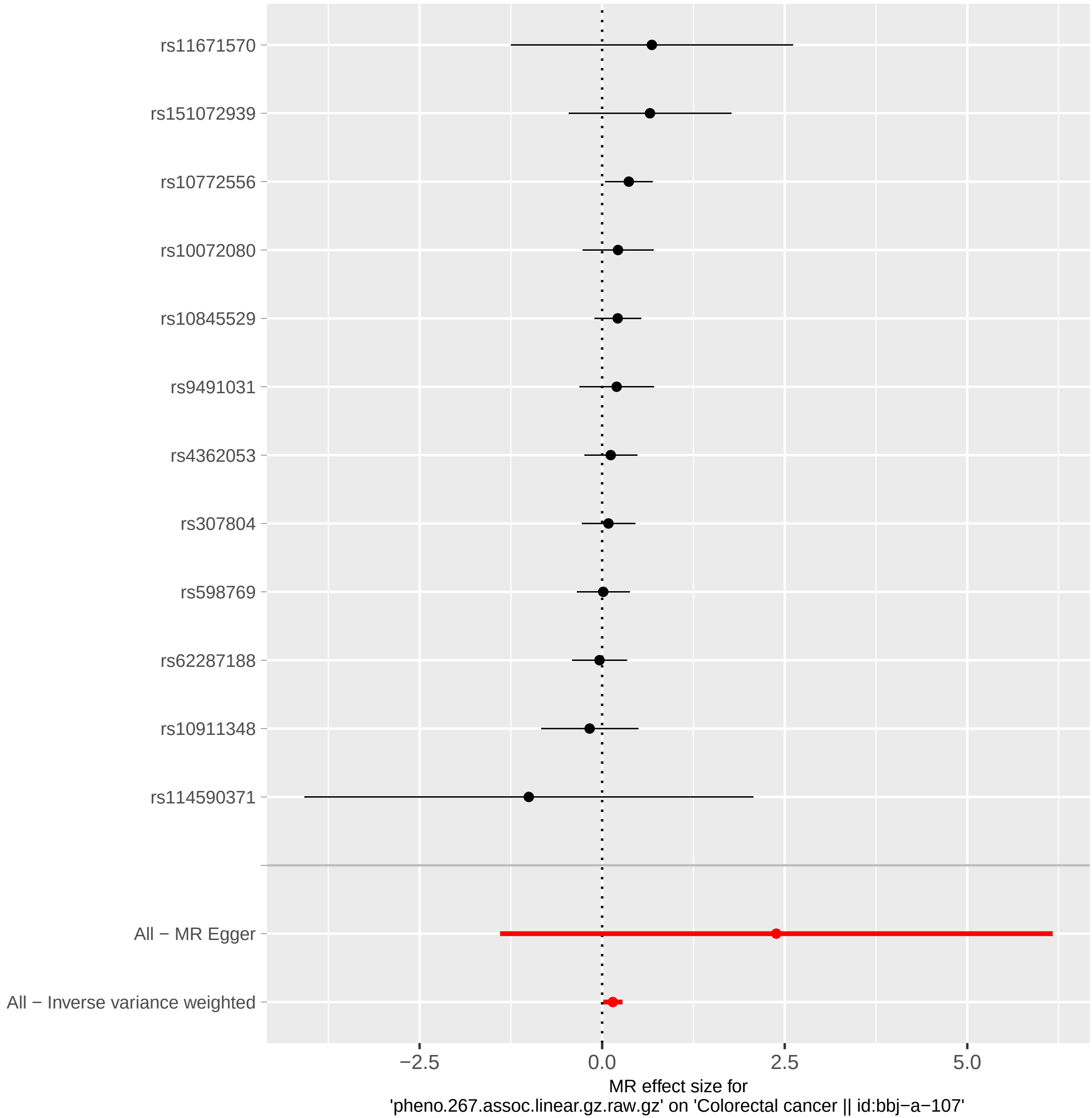
rs11671570
rs151072939
rs10772556
rs10072080
rs10845529
rs9491031
rs4362053
rs307804
rs598769
rs62287188
rs10911348
rs114590371
All − MR Egger
All − Inverse variance weighted
−2.5
0.0
2.5
5.0
MR effect size for
'pheno.267.assoc.linear.gz.raw.gz' on 'Colorectal cancer || id:bbj−a−107'
